# Growth-Phase-Dependent Shift in GABA Biosynthetic Pathways Under Temperature Stress in *Isochrysis zhanjiangensis*

**DOI:** 10.3390/microorganisms14092014

**Published:** 2026-09-10

**Authors:** Jiansen Luo, Lin Zhang, Jichang Han, Yumeng Wang, Jiaxin Yu, Jingbo Fan, Lulu Wang, Jiayi Cao, Kehou Pan, Jilin Xu

**Affiliations:** 1 School of Marine Sciences, Ningbo University, Ningbo 315832, China; luojiansen5556@163.com (J.L.); wangyumeng38@gmail.com (Y.W.); 13216647931@163.com (J.Y.); 15137115357@163.com (J.F.); wanglulu@nbu.edu.cn (L.W.); caojiayi@nbu.edu.cn (J.C.); 2 College of Food Science and Engineering, Ningbo University, Ningbo 315211, China; hanjichang@nbu.edu.cn; 3 Laoshan Laboratory, Qingdao 266237, China; mnlkhpan@163.com

**Keywords:** *Isochrysis zhanjiangensis*, GABA, temperature stress, growth phase

## Abstract

Temperature stress is a major constraint on the productivity of microalgae used in aquaculture. γ-Aminobutyric acid (GABA) is well-established as a key player in the stress tolerance of higher plants, yet its role in microalgae remains largely unexplored. Here, we examined the effects of low (15 °C), optimal (25 °C), and high (35 °C) temperatures on the GABA shunt in *Isochrysis zhanjiangensis* during the initial and mid-exponential growth phases. The results demonstrated that temperature stress significantly inhibited cell growth and photosynthetic efficiency (assessed by Fv/Fm and Fv’/Fm’), with soluble protein decreasing and soluble sugar accumulating. During the initial exponential phase, both low and high temperature stress triggered marked GABA accumulation, accompanied by coordinated increases in glutamate decarboxylase (GAD) and diamine oxidase (DAO) activities. Interestingly, the transcript levels of *IzGAD* and *IzDAO* decreased under these conditions, suggesting that GABA accumulation at this stage is predominantly governed by post-translational activation rather than transcriptional upregulation. Upon entry into the mid-exponential phase, a distinct phase-dependent shift in GABA biosynthetic regulation emerged. Under low temperature stress, GAD activity and *IzGAD* expression were both suppressed, whereas DAO activity and *IzDAO* transcripts increased significantly, indicating the transition to DAO-mediated GABA production as the dominant route. Under high temperature stress, both GAD and DAO activities increased, yet their corresponding gene transcription remained repressed, revealing a persistent asynchrony between enzyme activities and gene expression across both phases. Meanwhile, the expression of catabolic genes (*IzGABA-T*, *IzSSADH1*, and *IzSSADH2*) was consistently downregulated, further facilitating the net accumulation of GABA. Promoter analysis revealed multiple stress- and hormone-responsive cis-elements in these genes, implying a complex regulatory network. Collectively, our findings uncover a growth-phase-dependent reconfiguration of GABA biosynthetic pathways in *I. zhanjiangensis* under temperature stress. These insights provide a mechanistic basis for strain-specific temperature management in aquaculture applications.

## 1. Introduction

According to the World Meteorological Organization (WMO), global climate change is projected to raise the average surface temperature by 1.2–1.9 °C between 2025 and 2029 [1], accompanied by an increasing frequency of extreme temperature events [2,3]. Temperature stress adversely affects plant morphology, physiology, and biochemical processes, ultimately leading to reduced productivity [4]. Over the course of long-term evolution, plants have developed various strategies to perceive and cope with temperature stress [5]. Among these, the γ-aminobutyric acid (GABA) pathway has been widely documented for its role in enhancing plant stress resistance [6].

GABA is a four-carbon, non-proteinogenic amino acid that acts as a conserved signaling molecule across phylogenetically diverse organisms. In plants, GABA has been well-established as a key mediator of stress acclimation [6]. It contributes to a range of physiological processes, including the maintenance of carbon/nitrogen homeostasis, regulation of intracellular pH, modulation of endogenous phytohormone levels, and scavenging reactive oxygen species (ROS) [6]. Under normal conditions, GABA concentrations remain low; however, they rise rapidly and substantially in response to abiotic stressors [7]. For instance, GABA levels increased markedly in germinating *Vicia faba* under anoxic treatment [8] and in *Nicotiana tabacum* leaves within one hour of drought exposure [9]. Similarly, elevated temperatures were shown to enhance GABA accumulation in *Glycine max* L. Merrill [10] and *Oryza sativa* [11]. Furthermore, exogenous GABA application has been demonstrated to mitigate stress-induced damage and improve plant tolerance. Recent studies have indicated that foliar supplementation with GABA enhances cold tolerance in several species, such as apple [12] and *Diospyros kaki* [13].

GABA is primarily synthesized via two main pathways: the glutamate decarboxylation pathway and the polyamine degradation pathway [14]. The primary route for GABA biosynthesis is the glutamate decarboxylation pathway, catalyzed by glutamate decarboxylase (GAD), to produce GABA [6]. Alternatively, GABA can be generated via the polyamine degradation pathway, in which polyamines such as putrescine (Put), spermine (Spm), and spermidine (Spd) are oxidized by diamine oxidase (DAO) and polyamine oxidase (PAO). This process produces γ-aminobutyraldehyde (ABAL), which is then converted to GABA by aminoaldehyde dehydrogenase (AMADH) [6]. DAO is predominantly present in many dicotyledonous plants (e.g., legumes), whereas PAO is more common in monocotyledonous plants, like cereals [15]. However, there are exceptions; for instance, both DAO and PAO have been identified in soybeans [16]. In the focal organism of this study, *Isochrysis zhanjiangensis*, whose genome has been sequenced, only DAO is present. GABA catabolism begins with the conversion to succinic semialdehyde (SSA) through GABA transaminase (GABA-T). SSA is then further oxidized to succinate by succinic semialdehyde dehydrogenase (SSADH), entering the tricarboxylic acid cycle (TCA).

In vascular plants, the transcript abundance and enzymatic activities of the enzymes involved in GABA metabolism vary considerably across different developmental stages or tissue types. Panagiotis et al. [17] reported that both the abundance of transcripts and the enzymatic activity of PAO increased with the age of grape leaves. In *Arabidopsis*, the GAD gene family comprises five members (*AtGAD1* to *AtGAD5*) [18]. Among these, *AtGAD1* is primarily expressed in the roots, while *AtGAD4* and *AtGAD5* are mainly expressed in flowers. *AtGAD2* is ubiquitously expressed across various organs, whereas *AtGAD3* consistently maintains low expression levels in all tissues examined [18]. Additionally, various stress conditions differentially affect the activity and gene expression of these enzymes. In watermelon, *ClGAD3* expression was greatly increased after 8 days of salt stress, but was suppressed under drought stress for the same duration [19].

Microalgae represent a diverse group of microorganisms with broad applications, including food production, live feed, biofuel, and the extraction of bioactive compounds [20,21]. The primary cultivation systems for microalgae are open ponds and closed photobioreactors. Open cultivation systems are widely adopted due to their operational simplicity and low construction costs [20]. However, these systems are highly susceptible to fluctuations in ambient temperature [22]. Temperature is a critical factor influencing microalgal growth, affecting photosynthetic performance, membrane fluidity, and antioxidant enzyme activity [23]. The optimal temperature for growth varies among species. For example, *Chlamydomonas reinhardtii* exhibits optimal growth at 27.5 °C [24], while *I. zhanjiangensis* grows best at 25 °C. Exposure to temperatures outside this optimal range can hinder microalgal growth [23]. For example, *Phaeodactylum tricornutum* typically grows best between 10 °C and 20 °C. When cultivated at 30 °C for 72 h, its cell density reached only 42.85% of that observed in the control group maintained at 18 °C [25].

Compared to the extensive research on GABA in higher plants, studies on GABA in microalgae remain limited. To date, only a few reports have investigated the effects of exogenous GABA on microalgal growth, lipid production, and antioxidant enzyme activities [26]. Notably, GABA has been unequivocally demonstrated in higher plants to participate in stress-responsive processes, encompassing antioxidant defense, calcium signaling, and the maintenance of carbon-nitrogen homeostasis. *I. zhanjiangensis*, which belongs to the phylum Haptophyta, the family Isochrysidaceae, and the genus *Isochrysis*, is recognized for its rapid growth and high nutritional value [27], making it a commonly used live feed in aquaculture [28], and exhibits marked reductions in growth rate, biomass yield, and nutritional quality when exposed to temperature stress. Given the escalating frequency of extreme thermal events, elucidating the adaptive mechanisms that govern thermotolerance in this microalga is of paramount importance for stabilizing aquaculture production. While GABA has been extensively documented as a stress-protective metabolite and signaling molecule in higher plants, its functional roles and regulatory networks in microalgae remain largely unexplored, in particular, the pathway hierarchy between DAO and PAO for GABA biosynthesis, as well as their growth-phase-dependent modulation under thermal stress. Addressing this knowledge gap will not only enhance our fundamental understanding of stress physiology in microalgae, but also provide a theoretical basis for microalgal cultivation in commercial aquaculture and for the genetic engineering of algal strains to improve their thermotolerance. Consequently, it is important to investigate the temperature stress tolerance of this species. In this study, we examined GABA synthesis and degradation in *I. zhanjiangensis* under three different temperatures: low (15 °C), optimal (25 °C), and high (35 °C). The aim is to clarify how GABA metabolism responds to temperature stress in this species.

## 2. Materials and Methods

### 2.1. Microalgal Strain Cultivation

The microalga *I. zhanjiangensis* was obtained from the Microalgae Collection Center at Ningbo University, China. It was cultivated in the NMB3# [29] liquid medium under controlled conditions. The cultures were maintained in an incubator at 25 °C under an illumination intensity of 100 μmol photons·m^−2^·s^−1^ with a 12 h light/12 h dark photoperiod, and shaken 3–5 times per day to avoid cell adhesion.

### 2.2. Experimental Design

Cells were cultured during the initial exponential (ini-) and mid-exponential (mid-) growth phases under three temperature conditions: optimal temperature (25 °C), low temperature (15 °C), and high temperature (35 °C). This experimental design yielded six distinct treatment groups: ini-CT (25 °C), ini-LT (15 °C), ini-HT (35 °C), mid-CT (25 °C), mid-LT (15 °C), and mid-HT (35 °C). Cell density and chlorophyll fluorescence parameters were measured at 0, 24, and 48 h. Algal samples were collected by centrifugation at 4000× *g* for 10 min, immediately frozen in liquid nitrogen and stored at −80 °C for subsequent biochemical analyses. These samples were analyzed for soluble protein, soluble sugar, and GABA content, as well as for the enzyme activities of GAD and DAO. The transcript levels of genes involved in GABA synthesis and degradation were also examined.

### 2.3. Algal Density and Physiological Parameter Determination

Algal cell density was estimated by using a hemocytometer. Chlorophyll fluorescence parameters (Fv/Fm and Fv’/Fm’) were measured using a PSI fluorometer (AquaPen-C, Photon Systems Instruments, Brno, Czech Republic) [30]. Soluble protein content was measured using a BCA Protein Assay Kit (Xin Saimei Biotechnology Co., Ltd., Suzhou, China), following the manufacturer’s instructions. Soluble sugar concentration was estimated using the anthrone-sulfuric acid colorimetric method [31]. GABA content, as well as GAD and DAO activities, were measured using commercial assay kits (Suzhou Grace Biotechnology Co., Ltd., Suzhou, China), strictly following the manufacturer’s protocols. All samples were analyzed in triplicate.

### 2.4. Gene Sequence Analysis

Five genes involved in GABA metabolism were identified from the *I. zhanjiangensis* genome and designated as *IzDAO* (accession number PX380373), *IzGAD* (accession number PX380374), *IzGABA-T* (accession number OQ570644), *IzSSADH1* (accession number OQ570645), and *IzSSADH2* (accession number PX380375). The exon–intron structures and promoter regions of these genes were predicted using TBtools v2.136. The molecular weights and theoretical isoelectric points of the corresponding proteins were estimated using Expasy (https://www.expasy.org/, accessed on 31 May 2026). Subcellular localization of the deduced proteins was predicted using Cell-pLoc 2.0 (http://www.csbio.sjtu.edu.cn/bioinf/Cell-PLoc-2/, accessed on 31 May 2026). Potential cis-acting elements, including low temperature response elements and heat shock elements, were identified using PlantCARE (https://bioinformatics.psb.ugent.be/webtools/plantcare/html/, accessed on 31 May 2026).

### 2.5. Analysis of Gene Transcription Levels

As described in Section 2.2, algal cells were collected at 0, 24, and 48 h under three different temperatures to analyze the transcriptional levels of genes involved in GABA synthesis and degradation. Based on a previous study [32], *GAPDH2* was selected as the reference gene. Primers were designed using Primer Premier 5.0 (Table S1). Total RNA was isolated using the SteadyPure Universal RNA Extraction Kit (Accurate Biotechnology (Hunan) Co., Ltd., Changsha, China). First-strand cDNA synthesis was performed using the Evo M-MLV Reverse Transcription Kit (Accurate Biotechnology (Hunan) Co., Ltd., Changsha, China). Quantitative real-time PCR (qPCR) was performed under the following conditions: initial denaturation at 95 °C for 30 s, followed by 40 cycles of 95 °C for 5 s, 60 °C for 30 s, and 95 °C for 15 s. Relative gene expression levels were calculated using the 2^−ΔΔCt^ method. All analyses were performed with three biological replicates and three technical replicates per sample.

### 2.6. Statistical Analysis

Statistical analyses were performed using one-way analysis of variance (ANOVA) followed by Duncan’s multiple-range test in SPSS 26, with significance defined as *p* < 0.05. All figures were generated using Origin 2021.

## 3. Results

### 3.1. Effect of Temperature on Growth

**Figure 1:**
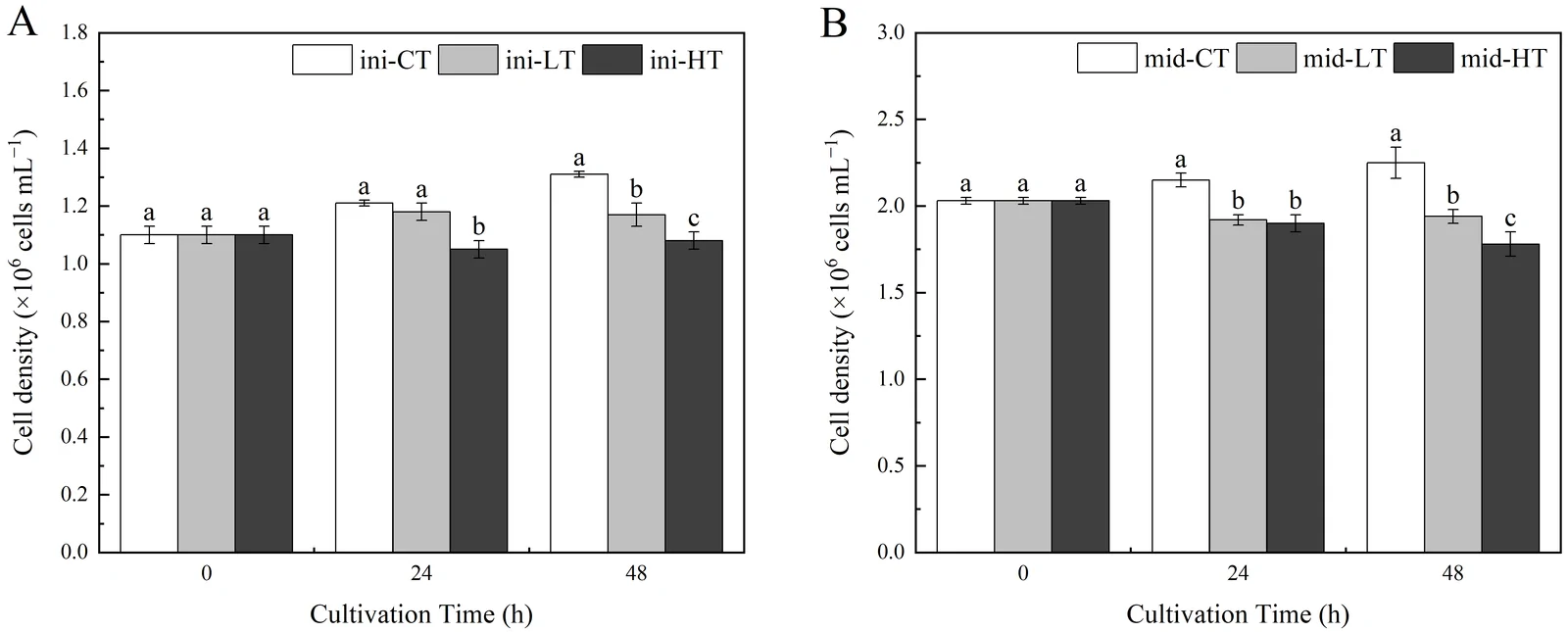
Growth of *I. zhanjiangensis* under different treatments. (**A**) Growth under different treatments during the initial exponential phases. (**B**) Growth under different treatments during the mid-exponential phases. ini, initial exponential growth phase; mid, mid-exponential growth phase; LT, temperature 15 °C; CT, temperature 25 °C; HT, temperature 35 °C. One-way ANOVA analysis followed by Duncan’s post hoc test was executed to estimate the differences. Different lowercase letters indicate significant differences (*p* < 0.05).

The growth dynamics of *I. zhanjiangensis* during the initial exponential phase were examined under different temperature conditions (Figure 1A). In the ini-CT group, cell density increased steadily. In contrast, the ini-LT group exhibited an initial rise followed by a decline, whereas a decline was observed in the ini-HT group. At 24 h, no significant difference in cell density was detected between the ini-LT and ini-CT groups (*p* > 0.05). In contrast, the cell density in the ini-HT group was 0.868 times that of the ini-CT group, indicating a significant reduction (*p* < 0.05). At 48 h, the cell densities in the ini-LT and ini-HT groups decreased to 0.893 and 0.824 times that of the ini-CT group, respectively, both significantly lower than that of the ini-CT group (*p* < 0.05).

When *I. zhanjiangensis* was subjected to temperature stress during the mid-exponential phase, distinct growth responses were also observed (Figure 1B). While the mid-CT group exhibited continuous growth, both the mid-LT and mid-HT groups showed declining cell densities over time. At 24 h, the cell densities in the mid-LT and mid-HT groups were 0.893 and 0.884 times that of the mid-CT group, respectively, both significantly lower than that of the mid-CT group (*p* < 0.05). At 48 h, the cell densities in the mid-LT and mid-HT groups further decreased to 0.862 and 0.791 times that of the mid-CT group, respectively, with both reductions being statistically significant (*p* < 0.05). Collectively, these findings demonstrated that both high and low temperature stresses suppress the growth of *I. zhanjiangensis* during the initial and mid-exponential phases, with high temperature exerting a more severe inhibitory effect.

### 3.2. Effect of Temperature on Chlorophyll Fluorescence

**Figure 2:**
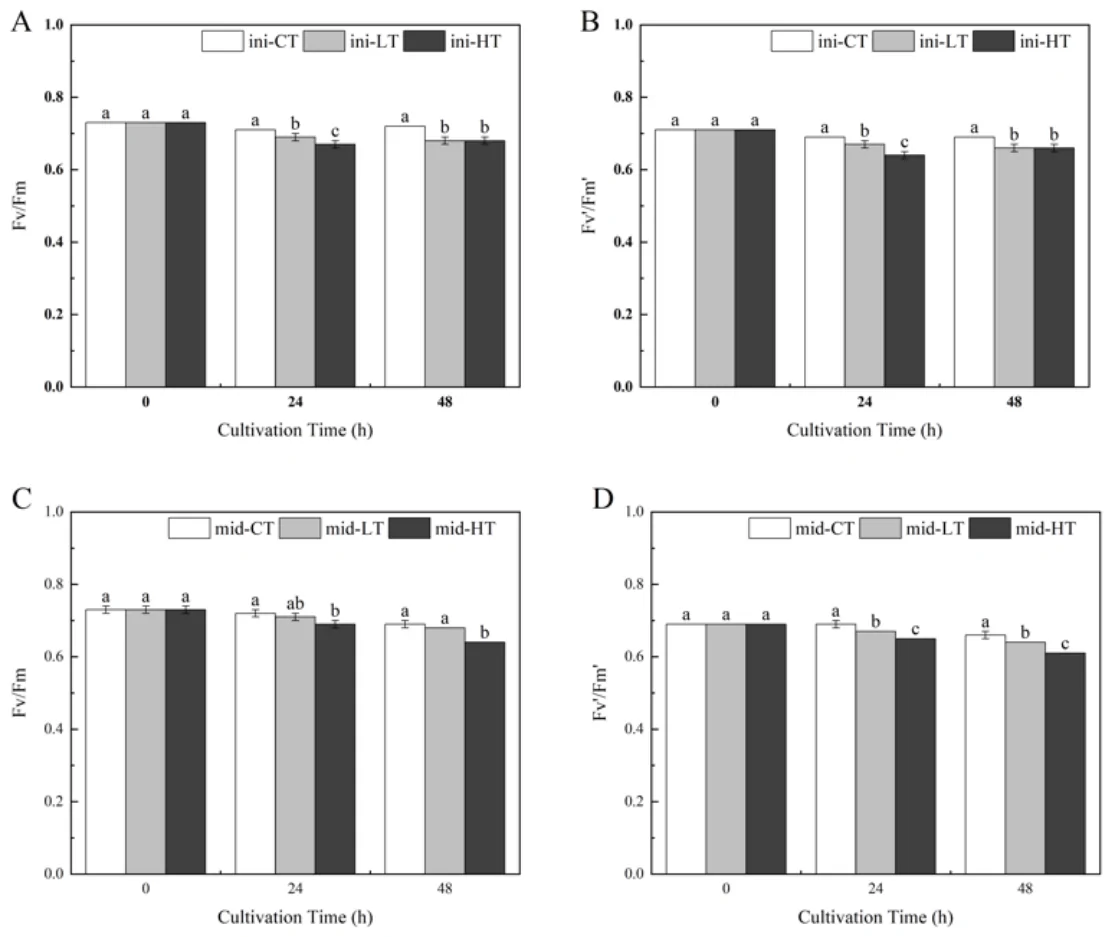
Chlorophyll fluorescence parameters (Fv/Fm and Fv’/Fm’) of *I. zhanjiangensis* under different treatments. (**A**) Fv/Fm under different treatments during the initial exponential phases. (**B**) Fv’/Fm’ under different treatments during the initial exponential phases. (**C**) Fv/Fm different treatments during the mid-exponential phases. (**D**) Fv’/Fm’ different treatments during the mid-exponential phases. ini, initial exponential growth phase; mid, mid-exponential growth phase; LT, temperature 15 °C; CT, temperature 25 °C; HT, temperature 35 °C. One-way ANOVA analysis followed by Duncan’s post hoc test was executed to estimate the differences. Different lowercase letters indicate significant differences (*p* < 0.05).

As shown in Figure 2A,B, when *I. zhanjiangensis* was cultured under different temperature conditions during the initial exponential phase, Fv/Fm values exhibited a declining trend over time, generally ranging between 0.67 and 0.73. After 24 h, the ini-HT group recorded the lowest Fv/Fm value. Significant differences were observed between the ini-HT and ini-CT groups, as well as between the ini-LT and ini-CT groups (*p* < 0.05). After 48 h, both the ini-LT and ini-HT groups showed a 5.56% decrease in Fv/Fm, significantly lower than that of the ini-CT group (*p* < 0.05). Fv’/Fm’ values generally ranged from 0.64 to 0.71. At both 24 and 48 h, the ini-LT and ini-HT groups showed significantly lower Fv’/Fm’ values than the ini-CT group (*p* < 0.05).

During the mid-exponential phase, a similar declining trend in Fv/Fm was observed across the mid-CT, mid-LT, and mid-HT groups, with values ranging from 0.64 to 0.73 (Figure 2C,D). After 24 and 48 h, significant differences in Fv/Fm were observed between the mid-LT and mid-CT groups, as well as between the mid-HT and mid-CT groups (*p* < 0.05). Fv’/Fm’ values ranged from 0.61 to 0.69. At both time points, the mid-LT and mid-HT groups showed significantly lower Fv’/Fm’ values than the mid-CT group (*p* < 0.05). These findings indicate that both high and low temperatures stresses negatively affect the photosynthetic efficiency of *I. zhanjiangensis* during the initial and mid-exponential growth phases.

### 3.3. Effect of Temperature on Soluble Protein and Soluble Sugar

**Figure 3:**
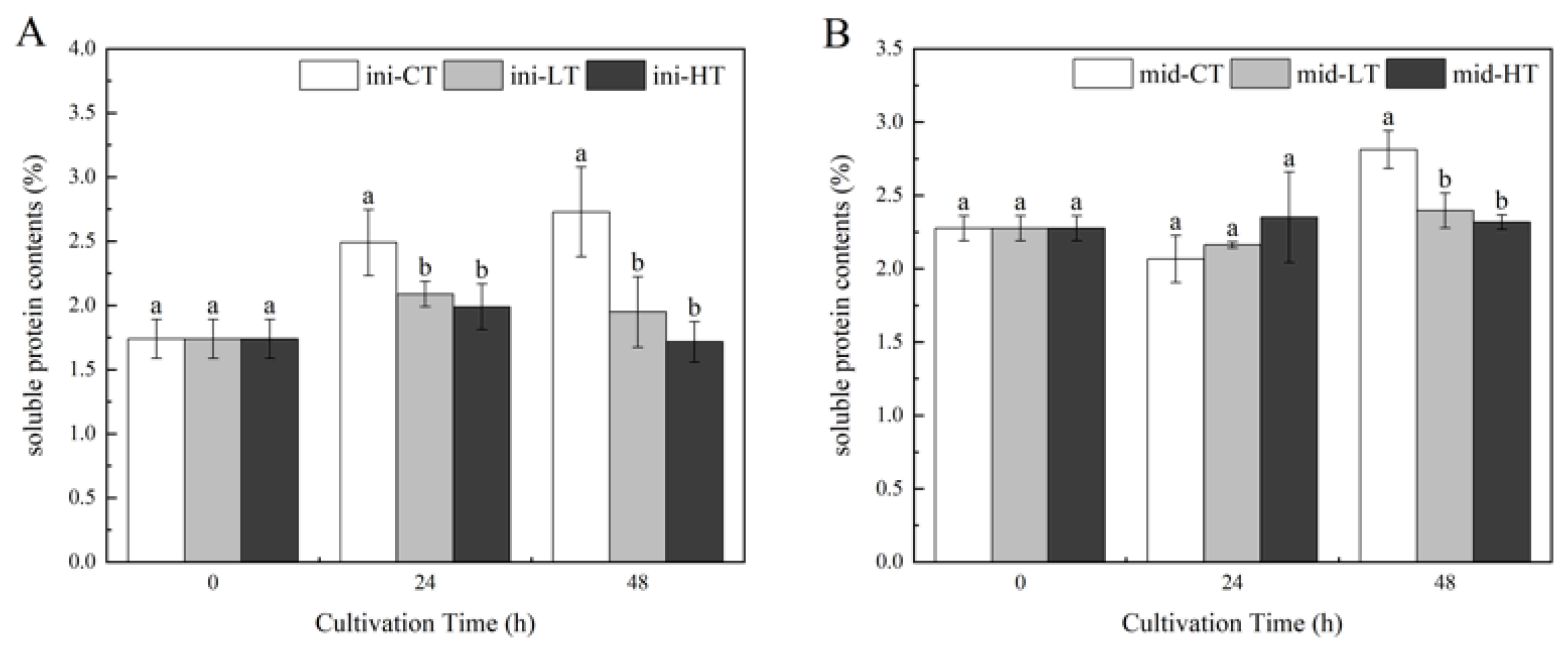
The soluble protein content of *I. zhanjiangensis* under different treatments. (**A**) The soluble protein content under different treatments during the initial exponential phases. (**B**) The soluble protein content under different treatments during the mid-exponential phases. ini, initial exponential growth phase; mid, mid-exponential growth phase; LT, temperature 15 °C; CT, temperature 25 °C; HT, temperature 35 °C. One-way ANOVA analysis followed by Duncan’s post hoc test was executed to estimate the differences. Different lowercase letters indicate significant differences (*p* < 0.05).

Figure 3A shows that the soluble protein levels in the ini-HT and ini-LT groups were significantly lower than those in the ini-CT group at both 24 and 48 h (*p* < 0.05). Notably, the response of algal cells during the mid-exponential phase differed from that observed during the initial exponential phase (Figure 3B). After 24 h, no significant differences were detected among the treatment groups (*p* > 0.05). However, by 48 h, the soluble protein content in the mid-LT and mid-HT groups was significantly lower than that in the mid-CT group (*p* < 0.05). Overall, the soluble protein content of *I. zhanjiangensis* was lower under both low and high temperature stress than under normal temperature conditions, irrespective of whether the cultures were in the initial or mid-exponential growth phase.

**Figure 4:**
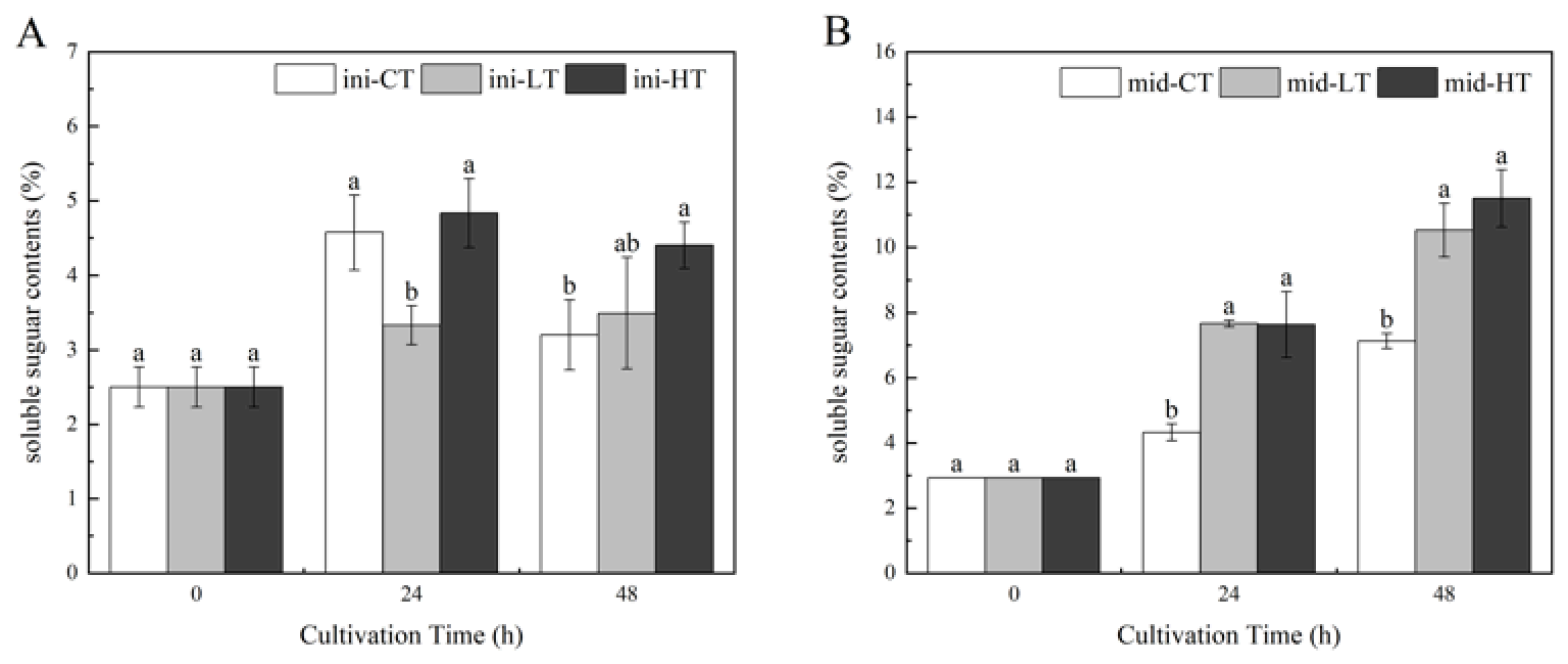
The soluble sugar content of *I. zhanjiangensis* under different treatments. (**A**) The soluble sugar content under different treatments during the initial exponential growth phase. (**B**) The soluble sugar content under different treatments during the mid-exponential growth phase. ini, initial exponential growth phase; mid, mid-exponential growth phase; LT, temperature 15 °C; CT, temperature 25 °C; HT, temperature 35 °C. One-way ANOVA analysis followed by Duncan’s post hoc test was executed to estimate the differences. Different lowercase letters indicate significant differences (*p* < 0.05).

For soluble sugar content (Figure 4A), in the initial exponential phase, a significant difference was observed between the ini-LT and ini-CT groups (*p* < 0.05), whereas no significant difference was detected between the ini-HT and ini-CT groups (*p* > 0.05). After 48 h, the soluble sugar content in the ini-HT group was significantly higher than that in the ini-CT group (*p* < 0.05), while the difference between the ini-LT and ini-CT groups was no longer significant (*p* > 0.05). During the mid-exponential phase, all groups exhibited an upward trend in soluble sugar content over time (Figure 4B). At both 24 h and 48 h, soluble sugar levels in the mid-LT and mid-HT groups were significantly higher than those in the mid-CT group (*p* < 0.05). Overall, under low and high temperature stress, the soluble protein content of *I. zhanjiangensis* generally decreased across all growth phases. In contrast, the soluble sugar content generally increased under the same stress conditions.

### 3.4. Effect of Temperature on GABA Concentration

**Figure 5:**
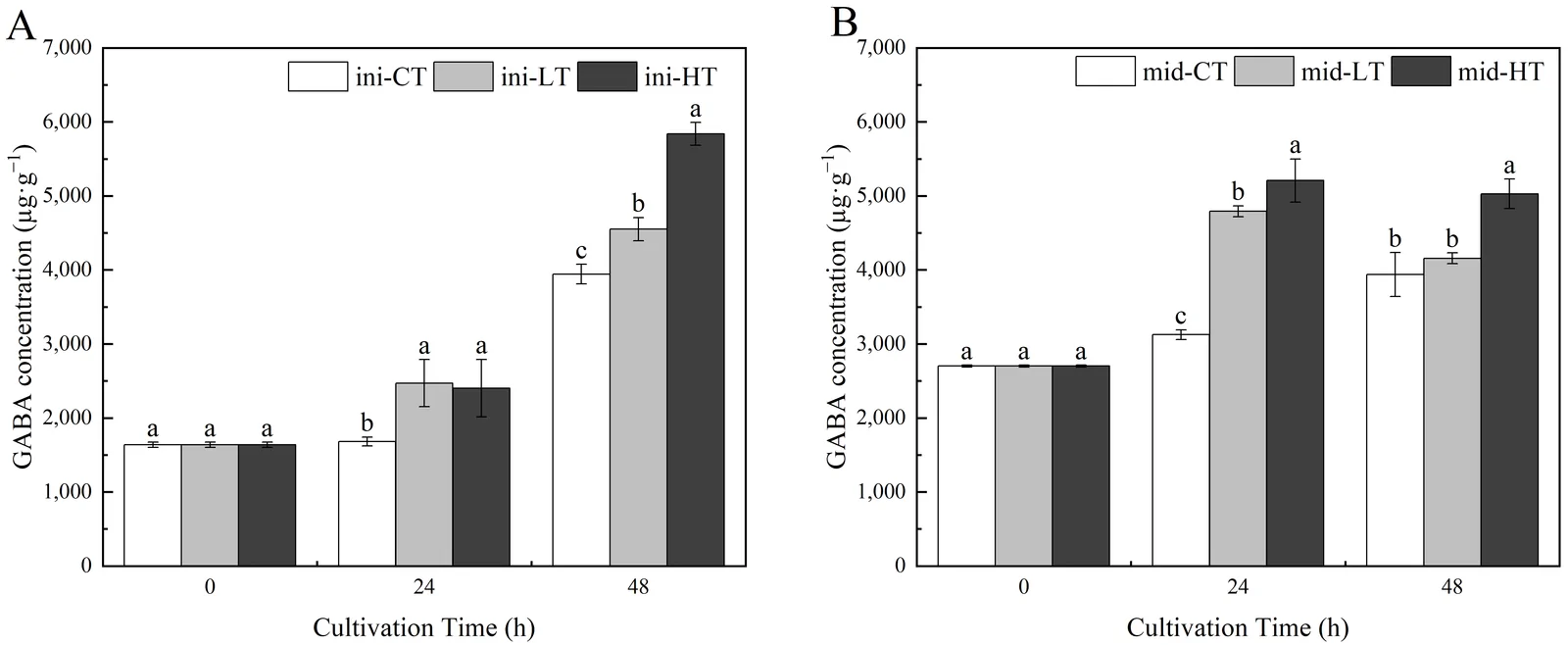
GABA concentration of *I. zhanjiangensis* under different treatments. (**A**) GABA concentration under different treatments during the initial exponential growth phase. (**B**) GABA concentration under different treatments during the mid-exponential growth phase. ini, initial exponential growth phase; mid, mid-exponential growth phase; LT, temperature 15 °C; CT, temperature 25 °C; HT, temperature 35 °C. One-way ANOVA analysis followed by Duncan’s post hoc test was executed to estimate the differences. Different lowercase letters indicate significant differences (*p* < 0.05).

As shown in Figure 5A, the GABA concentration in all groups increased over time during the initial exponential phase. After 24 h, GABA levels in the ini-LT and ini-HT groups were comparable and significantly higher than that in the ini-CT group (*p* < 0.05). By 48 h, the ini-HT group exhibited the highest GABA concentration, followed by the ini-LT group, with both significantly exceeding the ini-CT group (*p* < 0.05). The changes in GABA content during the mid-exponential phase are illustrated in Figure 5B. At 24 h, GABA levels in both stress groups were higher than in the mid-CT group, with the highest level observed in the mid-HT group (*p* < 0.05). After 48 h, the GABA concentration in the mid-LT group was comparable to that in the mid-CT group, whereas the mid-HT group maintained the highest level, significantly differing from both the mid-HT and mid-CT groups (*p* < 0.05). Temperature stress promotes the accumulation of GABA in *I. zhanjiangensis* during both the initial and mid-exponential growth phases.

### 3.5. Analysis of Genes Involved in GABA Synthesis and Degradation

Whole-genome sequencing of *I. zhanjiangensis* identified five candidate genes involved in GABA synthesis and degradation: *IzGAD*, *IzDAO*, *IzGABA-T*, *IzSSADH1*, and *IzSSADH2*. Table 1 summarizes the physicochemical properties of these genes. Their predicted protein lengths range from 233 to 792 residues. The theoretical isoelectric points (pI) range from 4.61 to 6.55, indicating that all encode acidic proteins (pI < 7.0). Subcellular localization predictions suggest their distribution in the chloroplast, cytoplasm, and peroxisome. The intron numbers for these genes are 6, 4, 0, 7, and 8, respectively, with corresponding exon numbers of 7, 5, 1, 8, and 9.

The 2000-bp promoter regions upstream of the five GABA-related genes were analyzed for putative cis-acting regulatory elements (Figure S1). These elements were classified into four major categories: those related to abiotic/biotic stress responses, phytohormone responses, light responses, and developmental processes. Notably, elements associated with abiotic and biotic stress responses—such as defense mechanisms against stress, anaerobic conditions, low temperatures, and drought—were identified in all five genes. Moreover, phytohormone-responsive elements were found to be widely distributed, with particular motifs responsive to auxin (IAA), salicylic acid (SA), abscisic acid (ABA), and methyl jasmonate (MeJA) being especially common. Collectively, these findings suggest that the five genes may play important roles in phytohormone signaling and stress response in *I. zhanjiangensis*.

### 3.6. Effect of Temperature on GABA Synthase Activity

**Figure 6:**
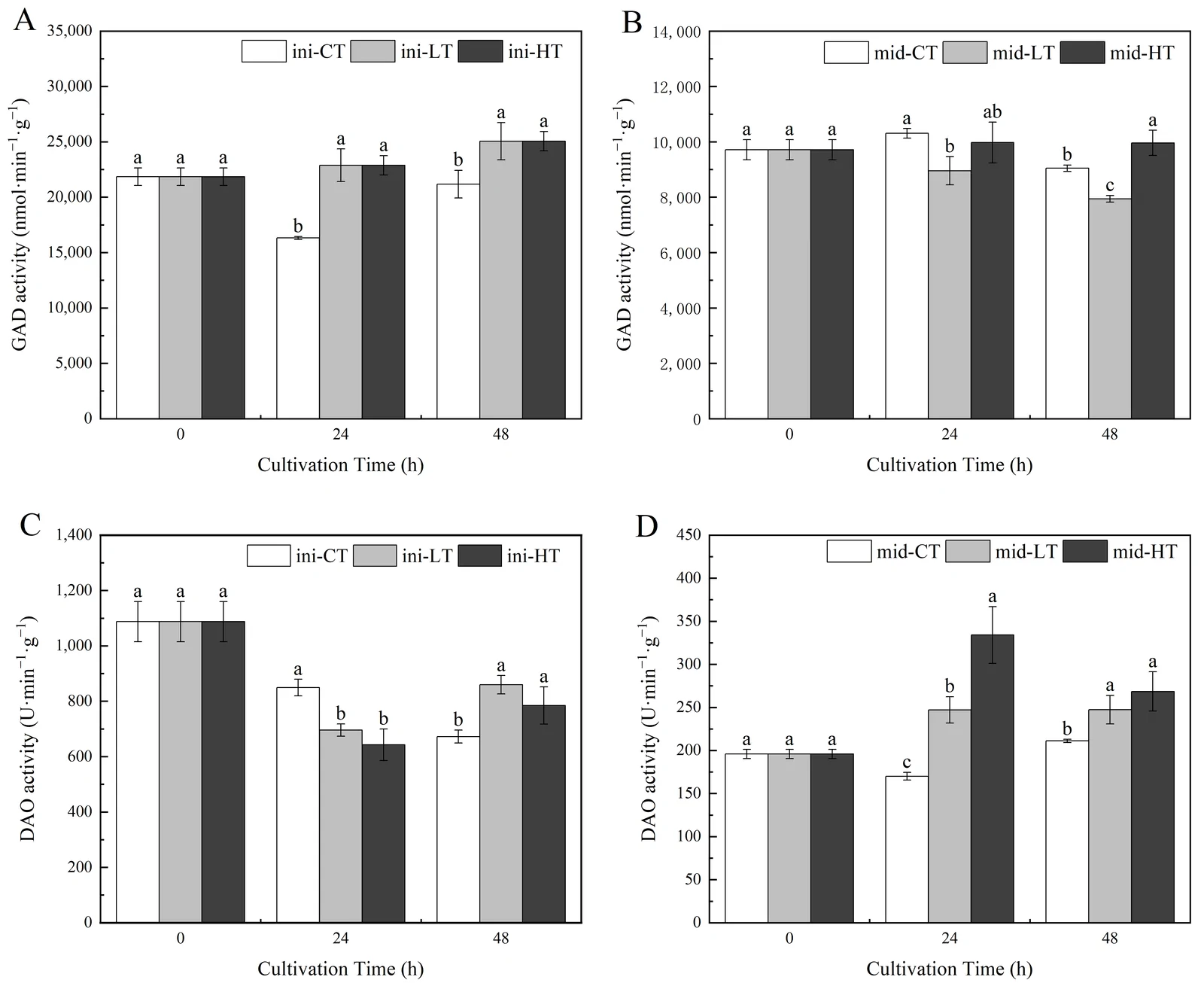
GAD activity and DAO activity of *I. zhanjiangensis* under different treatments. (**A**) GAD activity under different treatments during the initial exponential growth phase. (**B**) GAD activity under different treatments during the mid-exponential growth phase. (**C**) DAO activity under different treatments during the initial exponential growth phase. (**D**) DAO activity under different treatments during the mid-exponential growth phase. ini, initial exponential growth phase; mid, mid-exponential growth phase; LT, temperature 15 °C; CT, temperature 25 °C; HT, temperature 35 °C. One-way ANOVA analysis followed by Duncan’s post hoc test was executed to estimate the differences. Different lowercase letters indicate significant differences (*p* < 0.05).

During the initial exponential phase (Figure 6A), GAD activity in both stress groups was significantly different from that in the ini-CT group at 24 and 48 h (*p* < 0.05). As shown in Figure 6C, DAO activity in both stress groups was significantly different from that in the ini-CT group at 24 h (*p* < 0.05). By 48 h, DAO activity in these groups had increased substantially compared to the control (*p* < 0.05). These results indicate that both GAD and DAO were involved in the response of *I. zhanjiangensis* to high and low temperature stress during the initial exponential phase.

During the mid-exponential phase (Figure 6B), GAD activity in the mid-LT group was significantly different from that in the mid-CT group at 24 h (*p* < 0.05). However, no significant difference was detected between the mid-HT and mid-CT groups (*p* > 0.05). By 48 h, GAD activity in the mid-HT group was significantly higher than that in the mid-CT group (*p* < 0.05), while it was significantly lower in the mid-LT group (*p* < 0.05). In Figure 6D, DAO activity in the mid-LT and mid-HT groups was significantly higher than that in the mid-CT group at both 24 and 48 h, (*p* < 0.05). For *I. zhanjiangensis* at the mid-exponential growth phase, under low-temperature stress, GABA accumulation is attributable to suppression of the GAD pathway, with DAO playing the predominant role. Under high-temperature stress, GABA accumulation results from the synergistic action of both DAO and GAD.

### 3.7. Effect of Temperature on the Transcript Levels of GABA Synthesis and Degradation Genes

**Figure 7:**
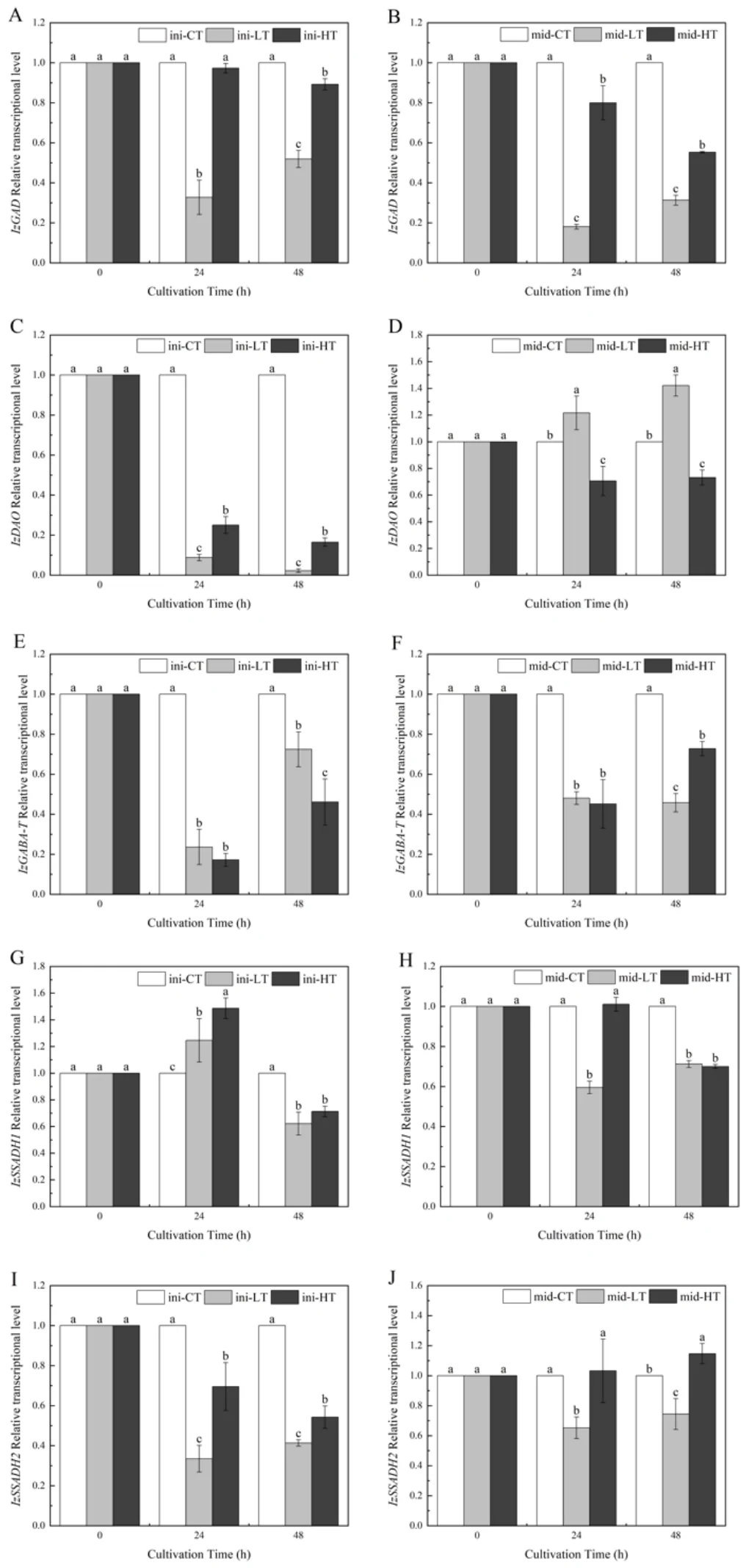
Relative transcription levels of *IzGAD*, *IzDAO*, *IzGABA-T*, *IzSSADH1*, and *IzSSADH2* under different treatments. (**A**) Relative transcription levels of *IzGAD* under different treatments during the initial exponential growth phase. (**B**) Relative transcription levels of *IzGAD* under different treatments during the mid-exponential growth phase. (**C**) Relative transcription levels of *IzDAO* under different treatments during the initial exponential growth phase. (**D**) Relative transcription levels of *IzDAO* under different treatments during the mid-exponential growth phase. (**E**) Relative transcription levels of *IzGABA-T* under different treatments during the initial exponential growth phase. (**F**) Relative transcription levels of *IzGABA-T* under different treatments during the mid-exponential growth phase. (**G**) Relative transcription levels of *IzSSADH1* under different treatments during the initial exponential growth phase. (**H**) Relative transcription levels of *IzSSADH1* under different treatments during the mid-exponential growth phase. (**I**) Relative transcription levels of *IzSSADH2* under different treatments during the initial exponential growth phase. (**J**) Relative transcription levels of *IzSSADH2* under different treatments during the mid-exponential growth phase. ini, initial exponential growth phase; mid, mid-exponential growth phase; LT, temperature 15 °C; CT, temperature 25 °C; HT, temperature 35 °C. One-way ANOVA analysis followed by Duncan’s post hoc test was executed to estimate the differences. Different lowercase letters indicate significant differences (*p* < 0.05).

As shown in Figure 7A, after 24 h, the *IzGAD* transcript level in the ini-LT group decreased by 67.22%, significantly lower than that in the ini-CT group (*p* < 0.05). By 48 h, the *IzGAD* transcript levels in both the ini-LT and ini-HT groups were significantly reduced relative to the ini-CT group (*p* < 0.05). From Figure 7B, the transcript levels of *IzGAD* in both stress groups were significantly lower than those in the mid-CT group at 24 and 48 h (*p* < 0.05).

During the ini-exponential phase (Figure 7C), the *IzDAO* transcript levels in the ini-LT and ini-HT groups were significantly downregulated at both 24 and 48 h (*p* < 0.05). In contrast, during the mid-exponential phase (Figure 7D), the *IzDAO* transcript level in the mid-LT group was significantly higher than that in the mid-CT group (*p* < 0.05), whereas it was significantly lower in the mid-HT group at both time points (*p* < 0.05).

In Figure 7E,F, the *IzGABA-T* transcript levels were significantly downregulated in both stress groups relative to their respective controls at both 24 and 48 h during both growth phases (*p* < 0.05).

As shown in Figure 7G, after 24 h, the *IzSSADH1* transcript levels in the ini-LT and ini-HT groups increased by 24.61% and 48.65%, respectively, significantly higher than those in the ini-CT group (*p* < 0.05). However, by 48 h, the transcript levels shifted from upregulation to downregulation, decreasing by 37.85% in the ini-LT group and 28.69% in the ini-HT group, and were significantly lower than those in the ini-CT group (*p* < 0.05). In Figure 7H, the transcript level of *IzSSADH1* in the mid-LT group was downregulated by 40.46% at 24 h, significantly lower than the mid-CT group (*p* < 0.05). At 48 h, transcript levels in both stress groups were significantly lower than those in the mid-CT group (*p* < 0.05).

Figure 7I illustrates that the transcript levels of *IzSSADH2* in the ini-LT and ini-HT groups were significantly lower than those in the ini-CT group at both 24 and 48 h (*p* < 0.05). In Figure 7J, the *IzSSADH2* transcript level in the mid-LT group was downregulated and significantly different from that in the mid-CT group at 24 h (*p* < 0.05). At 48 h, the transcript level in the mid-HT group was significantly higher than that in the mid-CT group (*p* < 0.05), whereas the mid-LT group remained significantly downregulated (*p* < 0.05).

## 4. Discussion

In recent years, the increasing frequency of extreme temperature events driven by global climate change has posed significant challenges to microalgal cultivation and productivity. GABA acts as both a stress metabolite and a signaling molecule, and has been shown to alleviate temperature stress in diverse organisms [6]. However, despite its well-documented roles in higher plants, the function of GABA in enhancing stress tolerance in microalgae remains largely unexplored. This study aims to characterize the dynamic changes in GABA levels under temperature stress in *I. zhanjiangensis*, a microalga recognized for its potential as a high-quality feed source in aquaculture.

Temperature directly affects photosynthetic efficiency by modulating the activity of enzymes involved in photosynthesis [33]. In algae, photosynthesis relies on the coordinated function of multiple components: photosystem I (PSI), photosystem II (PSII), the CO_2_ reduction pathway, photosynthetic pigments, and the electron transport system [34]. Impairment of any of these components can compromise photosynthetic performance [35]. Chlorophyll fluorescence technology has been widely used to analyze the kinetic characteristics of photosynthesis [36]. The maximum photochemical quantum yield of PSII (Fv/Fm) remains stable under non-stress conditions but decreases significantly under stress. The effective photochemical quantum yield (Fv’/Fm’) also shows a similar decline [37]. In the present study, Fv/Fm and Fv’/Fm’ of *I. zhanjiangensis* were inhibited under high and low temperature stress during the initial and mid-exponential growth phases, with more pronounced inhibition observed under high temperature. This finding aligns with the results of *Isochrysis galbana* [38] and *Arthrospira platensis* [39], indicating that the efficiency of light energy capture by the PSII reaction centers was reduced under high and low temperatures.

Environmental stress disrupts the osmotic balance within plant cells, causing dynamic changes in osmoregulatory substances. Among these, soluble proteins and soluble sugars contribute to part of the osmotic regulation in cells. Soluble proteins participate in stress responses by perceiving external signals via stress transduction pathways and subsequently synthesizing defensive and protective compounds to alleviate stress-induced damage [40]. Soluble sugars function as stress-induced metabolites that help stabilize intracellular osmotic pressure in response to external fluctuations [41]. The accumulation patterns of soluble proteins and soluble sugars under temperature stress vary considerably among species, depending on their specific physiological tolerances. Under low temperature stress, *Acer fabri* leaves exhibit a synergistic increase in both soluble protein and soluble sugar content [42]. Exposure of *Paeonia ostii* to 40 °C for seven days resulted in reductions in soluble protein and soluble sugar by 36.2% and 68.1%, respectively [43]. Similarly, a 24 h low temperature treatment led to decreased soluble protein and increased soluble sugar levels in ginseng roots [44]. In maize leaves, soluble protein and soluble sugar content increased with rising temperatures from 32 °C to 38 °C, peaked at 38 °C, and declined thereafter [45]. In this study, *I. zhanjiangensis* responded to temperature stress by decreasing soluble protein content while increasing soluble sugar content during both the initial and mid-exponential phases.

GABA has been shown to play a crucial role in temperature stress tolerance across diverse species. In wheat and barley, low temperature stress upregulated GABA metabolic genes, facilitating the rapid conversion of glutamate (Glu) to GABA [46]. Similarly, cold treatment in *Dimocarpus longan* Lour [47] and heat stress in creeping bentgrass [48] significantly increased the GABA levels. The application of exogenous GABA enhanced thermal tolerance in plants by reducing oxidative damage, activating signaling pathways, and promoting the production of stress-resistant metabolites. For instance, under high temperature stress, exogenous GABA alleviated oxidative stress in *Gracilaria lemaneiformis* by enhancing antioxidant enzyme activity, activating heat shock proteins, and promoting calcium dependent protein kinase (CDPK) signaling pathways [49]. In tea plants exposed to cold stress, exogenous GABA elevated endogenous GABA levels, which in turn enhanced the production of various metabolites, including polyamines (PAs), Glu, and anthocyanins, while also optimizing the antioxidant system, improving photosynthetic efficiency, and promoting carbon-nitrogen cycling, ultimately contributing to improved chilling tolerance [50]. In *Arabidopsis thaliana*, GABA concentrations increased in response to temperature stress, with a more pronounced rise under heat stress compared to cold exposure [51]. Under nitrogen deprivation, low temperature, and UV, the intracellular GABA content in *Synechocystis* sp. PCC 6803 significantly increased [52]. A similar trend was observed in *I. zhanjiangensis* during the initial and mid-exponential phases in this study, further highlighting the conserved role of GABA in the temperature stress response of microalgae.

GAD and DAO are key enzymes involved in GABA biosynthesis, and their activities have been shown to increase significantly under various stress conditions [53]. For example, low temperature treatment enhanced GAD activity in mulberry leaves [54], whereas chilling storage raised DAO activity in zucchini [55]. Conversely, tea plants subjected to 7 h of anaerobic treatment exhibited a sharp decline in GAD activity alongside a continuous increase in DAO activity [56], suggesting that these enzymes are regulated by distinct signaling pathways. In the current study, during the initial exponential phase, *I. zhanjiangensis* exhibited synchronized increases in both GAD and DAO activities under temperature stress, indicating their synergistic involvement in promoting GABA accumulation. During the mid-exponential phase, however, the low temperature group showed a decrease in GAD activity accompanied by an increase in DAO activity. This suggests that the temperature stress response may vary across growth phases. Specifically, during the initial exponential phase, both the GAD and DAO pathways act synergistically to promote GABA accumulation. In contrast, under low temperature stress during the mid-exponential phase, the DAO pathway appears to predominate over the GAD pathway as the primary route for GABA synthesis. Under high temperature stress, the regulatory patterns of both pathways remained consistent with those observed during the initial exponential phase.

Plant hormones play a crucial role in regulating stress responses in plants [6]. For example, exogenous application of ABA has been shown to reduce oxidative damage caused by high temperatures in chickpeas [57]. Similarly, foliar spraying of MeJA at the booting stage effectively enhances antioxidant capacity and photosynthetic performance in rice, thereby mitigating heat-induced damage [58]. Under stress conditions, plant hormones do not act independently but rather through synergistic cross-talk to coordinately regulate stress responses [59]. In heat tolerant soybean varieties, both ABA and gibberellin (GA) levels increased simultaneously in buds under heat stress, whereas GA levels decreased in heat-sensitive lines. This suggests that thermotolerant genotypes may adjust their hormonal balance to better cope with thermal stress [60]. This viewpoint is further reinforced by the observation that exogenous application of MeJA following salt stress elevates endogenous ABA and SA levels in *Impatiens walleriana* [61]. Several studies have clarified the interactive regulation between GABA and plant hormones [6]. For instance, under salt stress, exogenous GABA has been shown to increase ethylene production and enhance the expression of the GPCR gene encoding an ABA receptor in *Caragana intermedia* [62]. In *Citrus sinensis*, GABA treatment promotes the accumulation of SA, ABA, and JA [63]. This treatment enhanced chilling tolerance, suggesting that hormonal signals play a role in modulating GABA-mediated antioxidant systems and energy metabolism. In the current study, several hormone-responsive cis-elements were identified in the promoter regions of GABA-related genes in *I. zhanjiangensis*, including elements responsive to IAA (TGA-element and AuxRR-core), SA (TCA-element), ABA (ABRE), and MeJA (CGTCA-motif and TGACG-motif). These findings suggest that GABA may interact with multiple phytohormonal signaling pathways to enhance thermotolerance in *I. zhanjiangensis* under temperature stress.

In plants, gene expression often follows distinct patterns that are associated with specific growth stages [64]. In tomatoes, three GAD-encoding genes have been characterized: *SlGAD2* and *SlGAD3* are highly expressed during early fruit development, whereas *SlGAD1* exhibits increased transcript levels during the fruit ripening stage [65]. GAD genes also exhibit tissue-specific expression patterns in cotton. Specifically, *GhGAD5* and *GhGAD10* are expressed across all tissues, while *GhGAD3* and *GhGAD8* are found primarily in anthers. *GhGAD4* shows high expression in stems, and *GhGAD9* is mainly expressed in petals [66]. Under cadmium stress, only *GhGAD6* was significantly upregulated in cotton roots, stems, and leaves. Silencing *GhGAD6* via VIGS technology increased sensitivity to cadmium stress, manifested as more severe oxidative damage [66]. However, the above studies are all confined to higher plants. Whether the expression of GABA synthesis related genes in microalgae exhibits growth phase-dependent dynamics under temperature stress has been rarely studied to date. In the present study, a growth-phase-dependent difference was observed in *I. zhanjiangensis*. During the initial exponential phase, GAD and DAO enzyme activity increased, while *IzGAD* and *IZDAO* transcript levels decreased, indicating that the GAD and DAO pathways serve as the primary route for GABA synthesis in response to temperature stress at this stage. In contrast, during the mid-exponential phase under low temperature stress, *IzGAD* transcript levels and GAD enzyme activity decreased, while *IzDAO* transcript levels and DAO enzyme activity increased. This indicates a shift in the regulatory strategy for GABA synthesis during the mid-exponential growth phase, wherein the DAO-mediated biosynthesis pathway takes precedence over the GAD pathway for GABA production. This shift likely reflects an adaptive strategy employed by *I. zhanjiangensis* to modulate its temperature stress response according to growth phase.

SSADH and GABA-T are key rate-limiting enzymes involved in GABA catabolism. Zhang et al. [67] identified 8 *GAD* genes, 2 *GABA-T* genes, and 1 *SSADH* gene based on quinoa transcriptomic data. Among these, *CqGAD8* and *CqGABA-T2* were found to play crucial roles in GABA accumulation during quinoa germination. Additionally, overexpression of the *MaSSADH* gene from banana in *Nicotiana benthamiana* leaves resulted in a significant increase in GABA content [68]. In this study, the expression patterns of GABA metabolism-related genes in *I. zhanjiangensis* exhibited growth-stage-specific regulation. During the initial exponential phase, the expression of *IzSSADH1*, *IzSSADH2*, and *IzGABA-T* was downregulated, while GAD enzyme activity increased, indicating reduced GABA degradation and enhanced synthesis, which collectively led to a significant rise in GABA concentration. During the mid-exponential phase, *IzSSADH2* expression was upregulated under high temperature stress, suggesting that its functional role may vary depending on stress type and developmental stage. Overall, *I. zhanjiangensis* appears to maintain a dynamic balance between GABA homeostasis and temperature stress response by initially activating the GAD pathway during the early exponential phase and subsequently shifting to the DAO pathway during the mid-exponential phase.

Despite the well-established role of GABA in the stress tolerance of higher plants, its function in microalgae has remained largely underexplored. In higher plants, genetic and physiological tools are relatively well-established, providing a solid foundation for functional studies of GABA. In contrast, many microalgal species still lack stable transformation systems and efficient genome editing tools [69], which greatly hampers the in-depth investigation of GABA metabolic pathways. With microalgae serving as sustainable sources of biofuels and live feed, they are increasingly being cultivated in large scale outdoor systems, where they inevitably encounter harsh environmental conditions such as extreme temperatures, high light intensities, and high salt stress. Recent efforts have begun to explore stress tolerance mechanisms in these organisms, yet most studies remain descriptive. Therefore, elucidating GABA mediated stress responses in microalgae will not only broaden our understanding of the functional diversity of GABA metabolism, but also provide a theoretical basis for leveraging GABA to enhance microalgal resilience to environmental stresses, ultimately guiding future strain improvement and cultivation strategies.

## 5. Conclusions

Our findings reveal that temperature stress suppresses cell growth and photosynthetic efficiency, and triggers significant GABA accumulation—but the underlying biosynthetic routes are not fixed; instead, they undergo a growth-phase-dependent switch. During the initial exponential phase, GABA accumulation is driven primarily by post-translational activation of both GAD and DAO, with little contribution from transcriptional upregulation. As cells enter the mid-exponential phase, however, a pathway reconfiguration occurs: under low temperature stress, the DAO pathway supplants GAD as the dominant GABA-synthesizing route, whereas under high temperature stress, both enzyme activities rise but their transcription remains repressed, indicating a persistent decoupling between activity and gene expression across thermal conditions. Concurrent downregulation of GABA catabolic genes (*IzGABA-T*, *IzSSADH1*, and *IzSSADH2*) further reinforces net GABA accumulation throughout both phases. Notably, promoter analyses revealed the enrichment of ABA-, MeJA-, SA-, and IAA-responsive cis-elements in GABA metabolism-related genes, suggesting that GABA may function within a broader phytohormone signaling network to coordinate temperature acclimation in this marine microalga. This study demonstrates growth-phase-dependent reconfiguration of the GABA biosynthetic pathway in *I. zhanjiangensis* under temperature stress, providing novel insights into abiotic stress adaptation in algal cultivation while also offering a viable strategy for improving microalgal thermotolerance via the genetic modulation of GABA metabolism.

## Figures and Tables

**Table 1:** Identification of genes involved in GABA metabolism.

Predicted Gene Name	CDS Length/bp	Protein Length/aa	Molecular Weight/kDa	pI	Subcellular Location	Exon Number	Intron Number
*IzGAD*	1668	555	61.62	6.55	chloroplast	7	6
*IzDAO*	2379	792	87.00	4.61	chloroplast	5	4
*IzGABA-T*	702	233	25.48	5.59	cytosol	1	0
*IzSSADH1*	1581	526	55.43	5.44	chloroplast	8	7
*IzSSADH2*	1713	570	59.36	6.43	peroxisome	9	8

## Data Availability

The original contributions presented in this study are included in the article. Further inquiries can be directed to the corresponding authors.

## Supplementary Materials

The following supporting information can be downloaded at: https://www.mdpi.com/article/10.3390/microorganisms14092014/s1, Figure S1: Cis-acting regulatory elements in promoters of *IzGAD*, *IzDAO*, *IzGABA-T*, *IzSSADH1* and *IzSSADH2*; Table S1: Names and sequences of primers for PCR.
