# Supplementary material for: Growth-Phase-Dependent Shift in GABA Biosynthetic Pathways Under Temperature Stress in Isochrysis zhanjiangensis
Source: Microorganisms. 2026 Sep 10;14(9):2014. doi: 10.3390/microorganisms14092014 (PMC13608987; doi:10.3390/microorganisms14092014)
Supplement: Supplementary file 1 [file microorganisms-14-02014-s001.zip › Supplementary Figure S1.pdf]

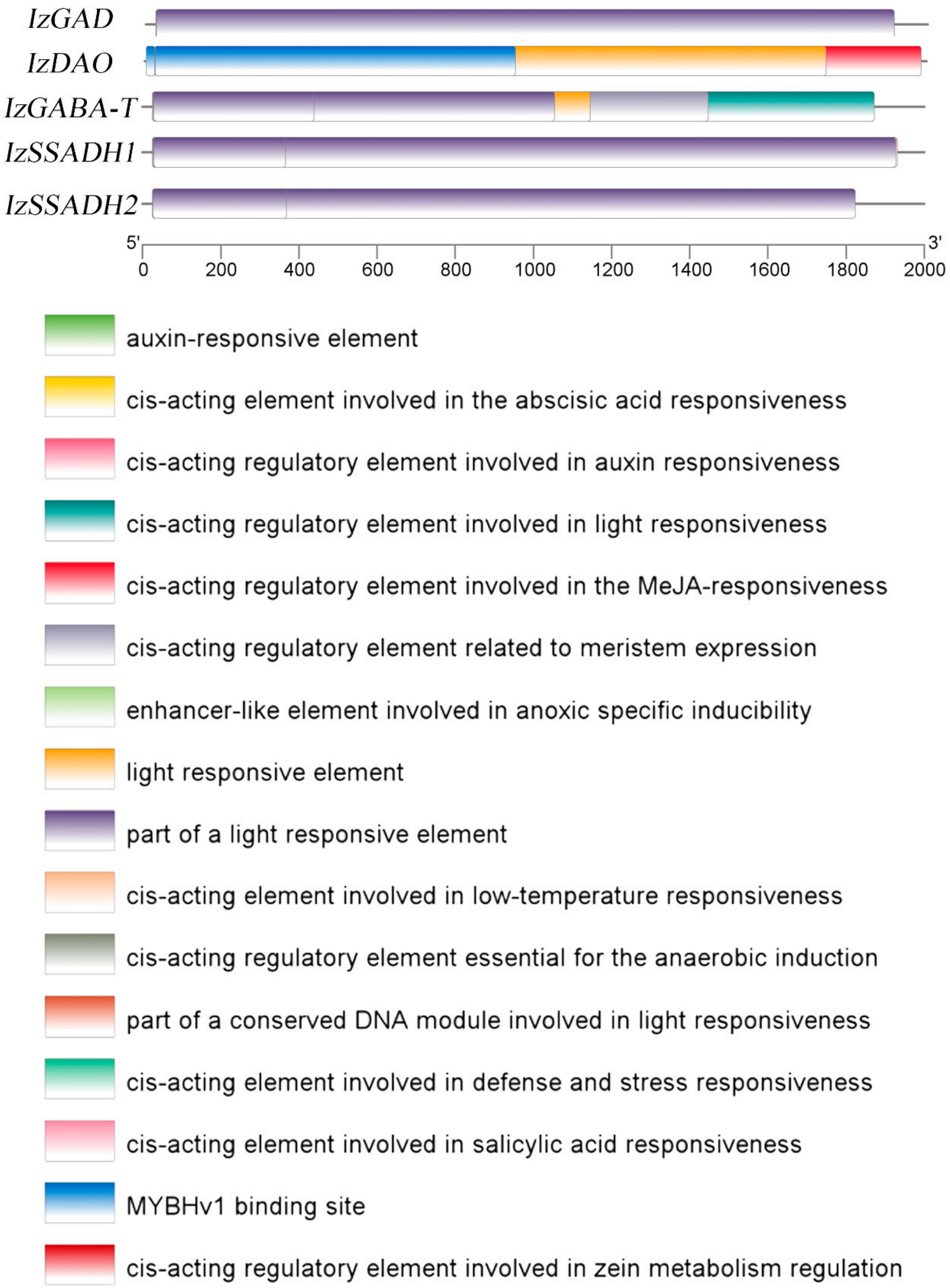

Figure S1. Cis-acting regulatory elements in promoters of *IzGAD, IzDAO, IzGABA-T, IzSSADH1* and *IzSSADH2*.
