# Supplementary material for: Growth-Phase-Dependent Shift in GABA Biosynthetic Pathways Under Temperature Stress in Isochrysis zhanjiangensis
Source: Microorganisms. 2026 Sep 10;14(9):2014. doi: 10.3390/microorganisms14092014 (PMC13608987; doi:10.3390/microorganisms14092014)
Supplement: Supplementary file 1 [file microorganisms-14-02014-s001.zip › Supplementary Table S1.pdf]

Table S1. Names and sequences of primers for PCR

Primer name	Primer sequence (5' – 3')
GAPDH2-F	CGTTGACTACGATACCGC
GAPDH2-R	CTTCTTAGCACCGCCCTG
IzDAO-F	ACAGAGGAGGAAGGGTA
IzDAO-R	TGGAGTGGATGTAAACG
IzGAD-F	ATCGTGTGGAGGCAGCG
IzGAD-R	GTTTGAGCAAAGGCGGG
IzGABA-T-F	TCACGCACACCCAGTAG
IzGABA-T-R	ATCGAGGCAGCCAAATA
IzSSADH1-F	AGAACGCACTACCCGCC
IzSSADH1-R	CCAAACCATTCGCAAAA
IzSSADH2-F	AATGAGCCGCAAGTCCACG
IzSSADH2-R	GACGAGCCCAACCAATCC
